# Professional quality of life, sleep disturbance and health among nurses: A mediation analysis

**DOI:** 10.1002/nop2.978

**Published:** 2021-07-22

**Authors:** Lena J. Lee, Leslie Wehrlen, Ya Ding, Alyson Ross

**Affiliations:** ^1^ Nursing Research and Translational Science National Institutes of Health Clinical Center Bethesda MD USA; ^2^ Office of Research Support and Compliance National Institutes of Health Clinical Center Bethesda MD USA

**Keywords:** burnout, compassion satisfaction, health, nurses, professional quality of life, secondary traumatic stress, sleep disturbance

## Abstract

**Aims:**

This study aimed to examine sleep disturbance as a mediator of the relationship between professional quality of life (compassion satisfaction, burnout, secondary traumatic stress) and health (physical and mental health) in nurses.

**Design:**

Descriptive, cross‐sectional study.

**Methods:**

Three hundred eighteen Registered Nurses completed a web‐based survey at the National Institutes of Health Clinical Center in the United States. Mediation analyses were conducted to test hypothesized relationships.

**Results:**

Nurses with higher levels of compassion satisfaction reported lower levels of sleep disturbance and better physical/mental health. Burnout and secondary traumatic stress were negatively associated with physical/mental health and positively associated with sleep disturbance. Sleep disturbance fully or partially mediated the relationships between professional quality of life and physical/mental health among nurses.

## 1 INTRODUCTION

Working as a nurse has many rewards, but the physical demands, irregular schedules, long work hours, and regular exposure to suffering and death can influence nurses’ professional quality of life (Flarity et al., 2013; Hinderer et al., 2014; Khamisa et al., 2015; Kim et al., 2015). Professional quality of life has emerged as a growing issue of interest among healthcare professionals (Kim et al., 2015). Professional quality of life refers to the positive (compassion satisfaction) and negative (compassion fatigue) aspects associated with working as a professional provider of care. Compassion satisfaction is the positive feeling gained from helping or caring for others and performing work‐related tasks well. Compassion fatigue refers to the negative consequences of working in a helping profession and is comprised of two aspects, burnout and secondary traumatic stress. Burnout is defined as the negative emotional reaction to external stressors associated within one's work environment (e.g., long working hours or heavy workloads). Secondary traumatic stress refers to the negative emotions and behaviours associated with exposure to work‐related traumatic stressful events (Stamm, 2010). Professional quality of life not only can impact nurses’ job performance (Stamm, 2010), but it can also directly influence their physical and mental health (Eanes, 2015; Fu et al., 2018; Khamisa et al., 2015). The relationship between workplace stress in nurses and sleep disturbances is well documented, and impaired sleep is considered a critically important issue facing nurses today (Caruso et al., 2019). There is good evidence that disturbed sleep contributes to significant physical and mental health issues for nurses (Caruso et al., 2019; Eanes, 2015; Kunzweiler et al., 2016; Perry et al., 2015; Sun et al., 2019). Nurses who are in poor physical and mental health as a result of workplace stress and workplace‐related sleep disturbances may represent a threat to patient safety (Caruso et al., 2019), as health problems in nurses can influence work efficiency and contribute to medical errors (Eanes, 2015; Zhang et al., 2017). Yet to date, few studies have examined the interrelationships between professional quality of life, sleep disturbance and health in nurses. Therefore, the aim of this study is to examine the mediating role of sleep disturbance on the relationship between professional quality of life and physical and mental health in nurses.

## 2 BACKGROUND

### 2.1 Professional quality of life and physical and mental health in nurses

Healthcare professionals in general are known to experience burnout and secondary traumatic stress, but nurses are particularly at risk (Kim et al., 2015). Numerous studies have examined the incidence of compassion satisfaction, burnout and secondary traumatic stress in nurses (Yilmaz & Üstün, 2018), but far fewer have examined the relationship between professional quality of life with physical and mental health in nurses. While compassion satisfaction appears to be protective of physical and mental health among nurses (Eanes, 2015; Fu et al., 2018; Khamisa et al., 2015), compassion fatigue is associated with declines in nurses’ health and quality of life (Fu et al., 2018). Nurses with high levels of compassion fatigue reported experiencing a variety of physical and psychological symptoms including headaches, gastric disturbances, depressive symptoms, anxiety and sleep disturbances (Fu et al., 2018; Khamisa et al., 2015). Khamisa et al. (2015) examined the relationships between work‐related stress, burnout, and job satisfaction with physical and mental health in 895 South African nurses and found that burnout was associated with deteriorations physical and mental health; in particular, burnout was the most influential factor predicting anxiety and insomnia.

### 2.2 Mediating role of sleep disturbance in the professional quality of life on health

The relationship between sleep disturbance and poor health outcomes is well established. Sleep disturbance contributes to not only a variety of chronic disease such as obesity, diabetes, cardiovascular disease and certain cancers (Dong et al., 2017; Eanes, 2015; Khormizi et al., 2018; Kunzweiler et al., 2016; Sun et al., 2019), but also to mental health problems such as depression (Eanes, 2015; Perry et al., 2015; Sun et al., 2019; Zhang et al., 2017). Nurses may be less likely than the general population to receive an adequate amount of quality sleep (Caruso, 2014; Eanes, 2015; Geiger‐Brown et al., 2012), likely because the nursing workplace often involves shiftwork. Shiftwork, particularly working nights or rotating shifts, is associated with circadian rhythm disruption that often leads to sleep deprivation, fatigue and diminished attention (Caruso, 2014; Imes & Chasens, 2019; Niu et al., 2011).

Thus, professional quality of life may impact a nurses’ sleep, and both sleep disturbances and professional quality of life may impact nurses’ health. However, to date no one has examined the interrelationships between professional quality of life, sleep disturbance and health in nurses. It is possible that sleep disturbance may affect the relationship between professional quality of life and health among nurses. Given the increased appreciation of nurses’ professional quality of life, most of the existing studies have primarily focused on burnout, and relatively little attention has been paid to the impact of compassion satisfaction and secondary traumatic stress on the health of nurses. Further, while sleep disturbances and compassion fatigue have been found to contribute to adverse health conditions in nurses, the mechanisms through which professional quality of life may affect nurses’ health have not been sufficiently investigated. Physical and mental health are complex concepts that likely are impacted by a variety of interrelated factors. Health risk factors, such as poor sleep, generally do not pose a singular, direct effect on health; rather, they tend to interact with other factors and conditions in multiple ways (Vitaliano et al., 2003). However, most research to date involving the health of nurses has focused on singular factors such as long work hours or burnout (Gómez‐García et al., 2016; Stimpfel et al., 2012).

### 2.3 Conceptual framework

**FIGURE 1:**
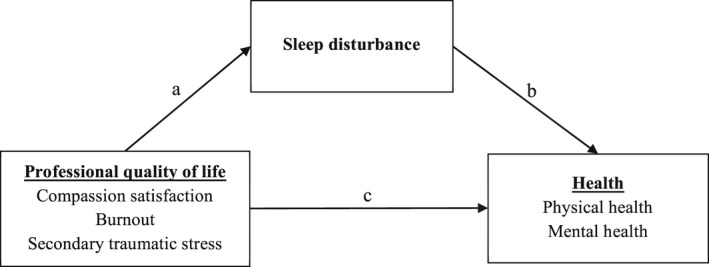
Theoretical model of sleep disturbance as the mediator between professional quality of life and health of nurses. Note: The dependent variable results from the direct effect of the independent variable (path c) as well as the mediating variable (path b). Variable mediating the independent variable can also be involved (path a)

This study is guided by the theoretical framework of Punnett et al. (2009), which suggests that the relationship between working conditions and employee health is multifaceted, with health behaviours such as sleep playing a role in the association between working conditions and health among employees (Figure 1). For example, negative working conditions such as exposure to workplace stress may contribute directly to an employee's physical and mental health. Workplace stress can also contribute to negative health behaviours such as poor sleep, which in turn may contribute to negative changes in physical and mental health. Likewise, positive working conditions, such as those whereby individuals have high levels of control and support, can have a protective effect on an individual's physical and mental health (Caruso et al., 2019; Punnett et al., 2009). However, these proposed pathways have not yet been examined empirically in nurses. Understanding underlying mechanisms accounting for the relationship between professional quality of life and health by assessing the interrelationships between professional quality of life, sleep and physical and mental health might lead to the development of more effective interventions to improve nurses’ health.

The objectives of the study were to explore the relationship among professional quality of life (compassion satisfaction, burnout, secondary traumatic stress), sleep disturbance and health (physical and mental health), and to examine the mediating role of sleep disturbance on the relationship between professional quality of life and health in nurses. Based on the proposed pathways described by Punnett et al. (2009), along with the results of previous studies, it was hypothesized that:

1. Professional quality of life (compassion satisfaction, burnout, secondary traumatic stress) would be associated with health (physical and mental health) among nurses,
2. Sleep disturbance would be associated with health among nurses, and sleep disturbance would mediate the relationship between professional quality of life and health.

## 3 METHODS

### 3.1 Study design, participants and setting

An anonymous cross‐sectional survey design was used to examine nurses’ participation in health‐promoting self‐care activities. Primary results of this study were published previously (Ross, Touchton‐Leonard, et al., 2019). All 1,363 Registered Nurses (RNs) working at the National Institutes of Health (NIH) Clinical Center in the United States, including those in direct patient care, administration, education, research and case management, were eligible to participate in this study. The NIH Clinical Center is a facility dedicated to clinical research with 200 inpatient beds, 93 outpatient day hospital stations and 15 outpatient clinics for research participants, most enrolled in early phase clinical trials (https://clinicalcenter.nih.gov/about/welcome/fact.html). The survey was launched during a 3‐week period in November 2016. Eligible participants were emailed study information along with the link to the anonymous online survey. The Strengthening the Reporting of Observational Studies in Epidemiology (STROBE) Statement: guidelines for reporting observational studies was used to guide the construction of this article (See Figure S1).

### 3.2 Sampling and survey procedure

The online survey was created using SurveyMonkey^®^ and delivered via email. The participants received a total of three emails during a 3‐week window. The initial email contained a letter from the Principal Investigator (PI) with a description of the study and a link to an anonymous online survey. One week after the first email was delivered, a reminder email was sent, followed a week later by a third final reminder and thank you. Announcements were also made at Nursing Practice Council, Leadership meetings and unit and clinic‐based staff meetings, where large groups of nurses routinely gathered.

### 3.3 Measures

#### 3.3.1 Professional quality of life

The Professional Quality of Life Scale (ProQOL) (Stamm, 2010) measures general job satisfaction within the timeframe of the last 30 days. The 30‐item ProQOL instrument includes three subscales: compassion satisfaction, burnout and STS. Each subscale consists of 10 questions, with each item on a 5‐point Likert scale, ranging from 1 (*never*) to 5 (*very often*). Each of the three subscales is scored by summing the 10 items, after appropriately reverse scoring is completed. The three subscales are evaluated based on pre‐determined cutoff scores (low ≤ 22; moderate = 23–41; high ≥ 42) (Stamm, 2010). This measure is valid and reliable in nursing populations (Flarity et al., 2013; Hegney et al., 2014). In this study, Cronbach alpha's values were compassion satisfaction = 0.916; burnout = 0.726; and secondary traumatic stress = 0.783.

#### 3.3.2 Physical and mental health

Physical and mental health were collected using the Patient‐Reported Outcomes Measurement Information System (PROMIS^®^) Global Health scale v1.0/1.1, which has exhibited good internal consistency reliability and strong construct validity across populations (Cella et al., 2010; Hays et al., 2009; Katzan & Lapin, 2018). The instrument consists of ten global health items that are used to calculate two component scores: global physical health and global mental health. Individual items for global physical health and global mental health are scored using a 5‐point Likert scale that ranges from 1 to 5, with higher scores indicative of better physical and mental health. The measures are standardized to a T‐score metric, with a mean of 50 and standard deviation of 10 that is centred around the general United States population. The measures offer clinically relevant physical and mental health thresholds (GPH: poor ≤ 35; fair = 36–42; good = 43–50; very good = 51–57; excellent ≥ 58, GMH: poor ≤ 29; fair = 30–40; good = 41–48; very good = 49–55; excellent ≥ 56) (Hays et al., 2015; HealthMeasures, 2020). In this study, the physical and mental health scales had Cronbach alpha coefficients of 0.704 and 0.850, respectively.

#### 3.3.3 Sleep disturbance

Sleep disturbance was measured by PROMIS^®^ Sleep Disturbance Short Form 4a, a valid and reliable measure that assesses self‐reported perceptions of sleep quality, sleep depth and restoration related to sleep (Buysse et al., 2010). The measure consists of four items that are scored on a 5‐point Likert scale. Higher scores reflect more sleep disturbance. A T‐score, normed to the general population with a mean of 50 and a standard deviation of 10, is calculated from the total sum score of the items. The measure provides clinically relevant sleep disturbance threshold (within normal limits ≤ 55; mild = 56–60; moderate = 61–69; severe ≥ 70) (HealthMeasures, 2020). The psychometric properties for the PROMIS^®^ sleep disturbance have been shown to be acceptable in nurses (Imes & Chasens, 2019; Kemper et al., 2015). In this study, the Cronbach alpha was 0.839.

### 3.4 Statistical analysis

The descriptive statistics (mean and frequencies) of the demographics and workplace characteristics were calculated to describe the sample. The assumptions (e.g., normality) of outcomes and the potential mediators were assessed prior to modeling. The Harman's single‐factor test was conducted to detect common method bias (Podsakoff et al., 2003). The results indicated that the common method variance was not a serious threat in this study. The correlations among ProQOL, sleep disturbance and health were computed. The mediation effect with path analysis was conducted to assess the effects of ProQOL on nurses’ health and how the effects were mediated by sleep disturbance. Based on existing evidence, age and gender were identified a priori as covariates and were controlled for in all the models. Multiple fit indices were used to determine whether the model was fit to the data: RMSEA < 0.08, CFI ≥ 0.95 and SRMR ≤ 0.08 (Hooper et al., 2008). The significance of the mediation effects was assessed using the non‐parametric, bias‐corrected 95% bootstrapped confidence interval (BCI) with 5,000 bootstrap replications (Mackinnon et al., 2004; Preacher & Hayes, 2008). The indirect effect is considered as significant if the zero is not included in the 95% BCI. All mediation analyses were conducted in Mplus Version 7.2 (Muthén and Muthén, (1998–2012)). Missing data in all models were estimated with the full information maximum likelihood (FIML) method used by Mplus. A *p* < .05 was considered significant.

### 3.5 Ethics

The NIH Clinical Center Office of Human Subjects Research Protections approved this study (OHSR #13263). Informed consent was implied if participants completed the online survey.

## 4 RESULTS

### 4.1 General characteristics of subjects

Of 1,363 RNs who received the survey, 335 RNs (24.6%) accessed the survey link and 318 (23.3%) completed the items needed for these analyses. Table 1 shows the descriptive characteristics of the participants. Age of the study sample ranged from 23 to 68 years (mean = 46.7, *SD* = 10.8 years), and the mean years of nursing practice was 19.2 (*SD* = 11.4) years. The large majority of the participants were female (91.1%), white (72.1%), married or partnered (72.3%), and well educated (92.8% held bachelor's degree or higher).

**TABLE 1:** Nurses’ health study descriptive characteristics (*N* = 318)

Characteristic	Mean (*SD*) range
Age in years	46.7 (10.8), 23–68
Years nursing practice	19.2 (11.4), 0–45
Sociodemographic characteristic	*n* (%)
Gender	
Female	266 (91.1)
Race	
White	207 (72.1)
Black/African American	32 (11.1)
Asian	23 ( 9.1)
Other^a^	22 ( 7.7)
Ethnicity	
Non‐Hispanic	267 (94.7)
Marital Status	
Married/partnered	211 (72.3)
Divorced/separated/never married/widowed	81 (27.7)
Education	
Diploma/Associate's degree	21 ( 7.2)
Bachelor's degree	151 (51.7)
Master's degree	107 (36.6)
Doctoral degree (PhD/DNP)	13 ( 4.5)
Workplace characteristics	*n* (%)
Employment Status	
Full Time	276 (86.8)
Part time/PRN/Per diem	42 (13.2)
Service Status	
Government/Civilian	292 (93.0)
Uniformed Service/PHS	22 ( 7.0)
Shifts worked	
Days only	224 (71.3)
Evenings only	6 ( 1.9)
Nights only	14 ( 4.5)
Rotating/Variable	70 (22.3)
Length of shift worked	
≤8 hr only	111 (35.0)
8–12 hr only	127 (40.1)
≥12 hr only	27 ( 8.5)
Variable	52 (16.4)
Primary Position	
Staff Nurse	124 (39.1)
Research Nurse/Study Coordinator/ Case Manager/Clinical Research Coordinator	95 (30.0)
Leadership (manager/supervisor, administrator, researcher, educator)	62 (19.5)
Advanced practice (NP, CNS, CRNA, CNM, etc…)	36 (11.4)
Professional quality of life	
Compassion satisfaction	40.4 ( 6.1), 19.0–50.0
Burnout	21.7 ( 5.2), 10.0–36.0
Secondary traumatic stress	20.4 ( 5.1), 11.0–36.0
Health outcomes	
Physical health	50.8 ( 7.6), 16.2–67.7
Poor	12 ( 4.0)
Fair	30 ( 9.9)
Good	76 (25.2)
Very good	105 (34.8)
Excellent	79 (26.2)
Mental health	48.4 ( 8.0), 21.2–67.6
Poor	4 ( 1.3)
Fair	32 (10.6)
Good	135 (44.6)
Very good	72 (23.8)
Excellent	60 (19.8)
Sleep disturbance	49.7 ( 7.4), 32.0–68.0
Within normal limits	233 (78.5)
Mild	41 (13.8)
Moderate	23 ( 7.7)

Abbreviations: CNM, Certified Nurse Midwife; CNS, Clinical Nurse Specialist; CRNA, Certified Registered Nurse Anaesthetist; n/a, not applicable; NP, Nurse Practitioner; PHS, Public Health Service; PRN, Pro Re Nata/As needed.

^a^ Other = unspecified (*n* = 14), multiracial (*n* = 7), American Indian or Alaska native (*n* = 2).

The mean scores for the level of compassion satisfaction, burnout and secondary traumatic stress were 40.4 (*SD* = 6.1), 21.7 (*SD* = 5.2), and 20.4 (*SD* = 5.1), respectively. In this sample, 166 participants (55.5%) had moderate levels of compassion satisfaction and 132 participants (44.1%) reported high levels of compassion satisfaction. The majority reported low levels of burnout (*n* = 186; 59.4%) and secondary traumatic stress (*n* = 215; 69.4%). Physical and mental health T‐scores on the PROMIS^®^ Global Heath Scale averaged 50.8 and 48.4, respectively, indicating that the study sample had a similar level of physical/mental health as the U.S. general population (Hays et al., 2009; HealthMeasures, 2020). Most of the nurses reported their physical health as good to excellent (*n* = 278, 86.2%), while 13.9% (*n* = 42) reported their physical health as poor. The large majority (*n* = 267, 88.2%) of the nurses reported their mental health as good to excellent, while 11.9% (*n* = 36) rated their mental health as fair or poor. Sleep scores on the PROMIS^®^ Sleep Disturbance Scale averaged 49.7, similar to levels found in the general population (HealthMeasures, 2020), although nearly a quarter of the nurses (*n* = 64, 21.5%) reported mild to moderate levels of sleep disturbance.

### 4.2 Correlations between ProQOL, sleep disturbance, and health

A number of statistically significant correlations were identified in the preliminary analysis among ProQOL, sleep disturbance, and physical and mental health (Table 2).

**TABLE 2:** Correlational analyses among professional quality of life, sleep and health

	1	2	3	4	5	6
1. Compassion satisfaction	‒					
2. Burnout	−0.610***	‒				
3. Secondary traumatic stress	−0.155**	0.552***	‒			
4. Sleep disturbance	−0.155**	0.371***	0.269***	‒		
5. Physical health	0.153**	−0.358***	−0.236***	−0.483***	‒	
6. Mental health	0.434***	−0.627***	−0.363***	−0.450***	0.602***	‒

Note

Professional quality of life = compassion satisfaction, burnout, secondary traumatic stress.

* *p* < .05.

** *p* < .01.

*** *p* < .001.

### 4.3 Associations between ProQOL, sleep disturbance and physical health

Table 3 summarizes direct and indirect effects of ProQOL, compassion satisfaction (Model 1a), burnout (Model 1b), secondary traumatic stress (Model 1c) and sleep disturbance on nurse's physical health.

**TABLE 3:** Direct and indirect effects of compassion satisfaction, burnout and secondary traumatic stress on physical health via sleep disturbance as a mediator

	Unstandardized Coefficient	SE	Bootstrap 95% CI
Model 1a			
Direct effect (c’ path)	0.057	0.042	−0.025, 0.137
Indirect effect (via mediator)			
Compassion satisfaction	0.053	0.022	0.011, 0.100
Model 1b			
Direct effect (c’ path)	−0.108	0.046	−0.276, −0.093
Indirect effect (via mediator)			
Burnout	−0.107	0.024	−0.159, −0.063
Model1c			
Direct effect (c’ path)	−0.093	0.044	−0.183, −0.013
Indirect effect (via mediator)			
Secondary traumatic stress	−0.090	0.021	−0.134, −0.052

Note

Age and sex controlled in all models.

Abbreviations: CI, Confidence Interval; SE, Standard Error.

#### 4.3.1 Model 1a

The total effect of compassion satisfaction on physical health was statistically significant (*β* = 0.142, *p* = .012). Increased compassion satisfaction was significantly associated with decreased sleep disturbance (*β* = −0.149, *p* = .017). The indirect (mediation) effect through the sleep disturbance was significantly significant (*β* = 0.068, bootstrap 95% confidence interval [BCI] = 0.011, 0.100). The direct effect of compassion satisfaction on physical health (*β* = 0.073, *p* = .171) was not statistically significant after adjusting for sleep disturbance. Thus, the association between compassion satisfaction and physical health was fully mediated by sleep disturbance.

#### 4.3.2 Model 1b

The total effect of burnout on physical health was statistically significant (*β* = −0.370, *p* < .001). The direct effect of burnout on the mediating variable of sleep disturbance was statistically significant (*β* = 0.360, *p* < .001). Sleep disturbance was significantly associated with physical health (*β* = −0.382, *p* < .001). In addition, burnout was directly associated with physical health (*β* = −0.232, *p* < .001). There was a statistically significant, indirect effect of sleep disturbance between burnout and physical health (*β* = −0.138, BCI = −0.159, −0.063). The results indicated that sleep disturbance played a partial mediator in the relationship between burnout and physical health.

#### 4.3.3 Model 1c

The total effect of secondary traumatic stress on physical health was statistically significant (*β* = −0.238, *p* < .001). Secondary traumatic stress had positively and significantly direct effect on sleep disturbance (*β* = 0.273, *p* < .001), while sleep disturbance was negatively and significantly associated with physical health (*β* = −0.428, *p* < .001). In addition, secondary traumatic stress was directly associated with physical health (*β* = −0.121, *p *= .033). The indirect effect through sleep disturbance was statistically significant (*β* = −0.117, BCI = −0.134, −0.052). The results indicated that sleep disturbance partially mediated the relationship between secondary traumatic stress and physical health.

### 4.4 Associations between ProQOL, sleep disturbance and mental health

Results from the mediation analyses that were conducted to identify direct and indirect effects of ProQOL, compassion satisfaction (Model 2a), burnout (Model 2b), secondary traumatic stress (Model 2c) and sleep disturbance on nurses’ mental health are shown in Table 4.

**TABLE 4:** Direct and indirect effect of compassion satisfaction, burnout and secondary traumatic stress on mental health via sleep disturbance as a mediator

	Unstandardized Coefficient	SE	Bootstrap 95% CI
Model 2a			
Direct effect (c’ path)	0.294	0.041	0.214, 0.377
Indirect effect (via mediator)			
CS	0.048	0.020	0.011, 0.090
Model 2b			
Direct effect (c’ path)	−0.439	0.042	−0.517, −0.355
Indirect effect (via mediator)			
BO	−0.072	0.017	−0.110, −0.044
Model 2c			
Direct effect (c’ path)	−0.208	0.046	−0.301, −0.121
Indirect effect (via mediator)			
STS	−0.085	0.020	−0.129, −0.049

Note

Age and sex controlled in all models.

Abbreviations: CI, Confidence Interval; SE, Standard Error.

#### 4.4.1 Model 2a

The total effect of compassion satisfaction on mental health was statistically significant (*β* = 0.418, *p* < .001). The direct effect of compassion satisfaction on the mediating variable of sleep disturbance was statistically significant (*β* = −0.151, *p* = .015). Sleep disturbance was significantly negatively associated with mental health (*β* = −0.386, *p* < .001). In addition, compassion satisfaction was directly associated with mental health (*β* = 0.359, *p* < .001). There was a statistically significant, indirect effect of sleep disturbance between compassion satisfaction and mental health (*β* = 0.058, BCI = 0.011, 0.090). Sleep disturbance partially mediated the relationship between compassion satisfaction and mental health.

#### 4.4.2 Model 2b

The total effect of burnout on mental health was statistically significant (*β* = −0.629, *p* < .001). Burnout was directly associated with the mediating variable of sleep disturbance (*β* = 0.362, *p* < .001). Sleep disturbance was significantly negatively associated with mental health (*β* = −0.244, *p* < .001). In addition, burnout was directly associated with mental health (*β* = −0.540, *p* < .001). The indirect pathways between burnout and mental health through sleep disturbance was statistically significant (*β* = −0.088, BCI = −0.110, −0.044). The results indicated that sleep disturbance partially mediated the relationship between burnout and mental health.

#### 4.4.3 Model 2c

The total effect of secondary traumatic stress on mental health was statistically significant (*β* = −0.360, *p* < .001). The direct effect of secondary traumatic stress on sleep disturbance was statistically significant (*β* = 0.276, *p* < .001) Sleep disturbance was negatively and significantly associated with mental health (*β *= −0.379, *p* < .001). In addition, secondary traumatic stress was directly associated with mental health (*β* = −0.255, *p* < .001). The indirect effect through the sleep disturbance was statistically significant (*β* = −0.105, BCI = −0.129, −0.049). The results indicated that sleep disturbance played a partial mediator in the relationship between secondary traumatic stress and mental health among nurses.

## 5 DISCUSSION

This study aimed to investigate the direct impact of professional quality of life on sleep disturbance and health, and the mediating role of sleep disturbance in the direct relationship among nurses. Our study findings revealed that nurses experiencing higher levels of compassion satisfaction were more likely to have better physical and mental health, while nurses with higher levels of burnout and secondary traumatic stress were more likely to experience poor physical and mental health. The association between professional quality of life and health in this population of nurses is consistent with previous research (Fu et al., 2018; Khamisa et al., 2015). Like the findings of Fu and colleagues (2015), all three dimensions of professional quality of life (compassion satisfaction, burnout, secondary traumatic stress) were significantly associated with physical and mental health in nurses. Like Khamisa et al. (2015), burnout was associated with worsened physical and mental health and insomnia, although our study went a step further and found that burnout, and most other aspects of professional quality of life, contribute both directly to physical and mental health, but also indirectly to physical and mental health via worsening sleep disturbances. The findings of this study may indicate that enhancing compassion satisfaction and reducing burnout and secondary traumatic stress may be particularly beneficial in improving the physical and mental health of nurses, especially those nurses who are experiencing sleep disturbances.

As anticipated, nurses in this study who reported sleep disturbances were more likely to exhibit poor physical and mental health. The findings of this study thereby confirm previous studies that established an association between sleep disturbance and health among nurses (Dong et al., 2017; Eanes, 2015; Khormizi et al., 2018; Kunzweiler et al., 2016; Perry et al., 2015; Sun et al., 2019; Zhang et al., 2017). More importantly, the present study adds new evidence to the literature by reporting that, after controlling for age and gender, sleep disturbance mediates the association between professional quality of life and nurses’ health. The findings therefore support the pathway proposed by Punnett's conceptual model (2009), which suggested a possible role of individual health behaviours (e.g., smoking, diet, exercise, sleep) in mediating the association between employees’ working conditions and general health. This study provided confirmation of this model by identifying a specific mediator of physical and mental health, sleep disturbance, in a relatively large sample of nurses. The findings of this study underscore the important role of sleep when trying to understand the mechanisms whereby professional quality of life impacts the physical and mental health of nurses. Further, the findings highlight the critical importance of addressing nurses’ professional quality of life when developing and supporting programmes that improve sleep quality in nurses.

This study provides new insights that the interrelationships between professional quality of life with physical and mental health in nurses are not necessarily straight forward and may involve a number of interrelating factors. This is not surprising, given that physical and mental health are complex concepts, and there likely are numerous pathways whereby professional quality of life, or other nursing workforce factors, may impact nurses’ health. If sleep mediates the relationship between professional quality of life and health in nurses, so might other health behaviours, such as exercise and nutrition, and these relationships are worthy of future research exploration. For example, nurses with high levels of compassion satisfaction, who derive a sense of pleasure and enjoyment from their jobs, may be more likely to exercise and/or to eat a healthy diet and therefore have better physical and mental health. Likewise, nurses with high levels of burnout and/or secondary traumatic stress may have less energy to exercise and/or may be more likely to consume foods high in sugar, fat and salt, all of which may then contribute to worsening physical and mental health. Our own past research showed that nurses with higher levels of compassion satisfaction indeed are more likely to exercise and consume a healthier diet (Ross, Yang, et al., 2019), but studies involving larger sample sizes are needed to more fully understand the complex pathways whereby professional quality of life directly and indirectly impacts nurses’ health.

Most past research examining professional quality of life in nurses has focused on the prevalence and dangers of compassion fatigue, mostly burnout, and to a lesser extent secondary traumatic stress. However, the importance of compassion satisfaction as a resilience factor for nurses should not be underestimated, as this study provides evidence that compassion satisfaction can be protective to the physical and mental health of nurses. Workplace factors that contribute to burnout and secondary traumatic stress, particularly factors such as shiftwork, long work hours, and/or the stress of working with very sick or dying patients, may be difficult if not impossible to rectify. However, it may be possible, and perhaps even easier, to design and implement effective interventions that increase a nurses’ enjoyment and the pleasure that they derive from their work. At the very least, future studies are needed to more fully understand the protective nature of compassion satisfaction to nurses’ health and well‐being, as there may be other pathways whereby compassion satisfaction contributes to improved physical and mental health than through improved sleep.

### 5.1 Implications for practice

Understanding of the interrelationships between professional quality of life, sleep and health is important because it may lead to the development of interventions that promote healthy sleep and improve nurses’ professional quality of life. Clearly, hospitals and healthcare providers need to be aware of the negative effects of burnout and secondary traumatic stress on nurses’ health and should encourage organizational initiatives that decrease burnout by providing nurses with higher levels of control and autonomy, access to resources for coping with traumatic experiences, and that improve workplace social support. Such initiatives would not only decrease burnout and secondary traumatic stress, but they likely would increase compassion satisfaction, the pleasure and satisfaction that nurses derive from their work. By improving nurses’ professional quality of life, healthcare leaders would also be improving nurses’ sleep, which potentially would reduce absenteeism, improve productivity, and reduce workplace injuries and/or medical errors. The positive impact of a well‐rested, healthy nursing workforce cannot be underestimated. In order to induce and sustain sleep or preserve alertness at work, nurses themselves must consider the priority they place on sleep. Nurse managers can provide health promotion education, including scheduling of bed‐time, regulating and timing caffeine, and improving the sleep environment. They can also advocate for employee screening for sleep disorders that provide referrals for nurses with impaired sleep. At the very least, nurse managers can educate nurses about the impact of burnout and secondary traumatic stress on sleep and health, and they can provide nurses with information on improving sleep hygiene to mitigate the effects of burnout and secondary traumatic stress on the health of nurses. The healthcare providers in the employee health department must have a systematic plan at all levels of the organization to recognize sleep‐related occupational health events, such as sleep complaints, drowsiness at work, and how work is organized to the advantage employees’ sleep.

### 5.2 Limitations/future directions

This study provides innovative information about the mediating role of sleep disturbance in the association between professional quality of life and health in nurses, but some limitations should be considered when interpreting the findings. The primary limitation was the use of a cross‐sectional design, which cannot provide information about causal relationships among variables. Studies utilizing longitudinal designs are needed in order to confirm causal relationships among professional quality of life, sleep disturbance, and physical and mental health of nurses. In addition, this study only recruited nurses from the NIH Clinical Center, a unique research hospital with a highly educated, all‐RN nursing workforce. Thus, the findings of this study may not be generalizable to other nursing populations. In the future, studies are needed that recruit nurses with different levels of education and who work in diverse medical settings in order to determine whether these findings can be replicated with other populations.

## 6 CONCLUSION

This study used a mediation analysis to contribute additional knowledge regarding the complexity of the interrelationships between professional quality of life, sleep disturbance and health in nurses. This study confirmed the importance of professional quality of life to nurses’ health and well‐being that had been found in other studies, and the relatively large sample size allowed for the novel inclusion of a mediator, sleep disturbance, as an additional pathway whereby compassion satisfaction, burnout and secondary traumatic stress influence the health of nurses. The findings support Punnett's conceptual model (2009), by confirming that there is a direct pathway between nurses’ professional quality of life and their physical and mental health, and that is pathway is mediated by sleep disturbance. It is important for healthcare organizations to protect the health of nurses by developing and supporting programmes that promote healthy sleep and improve nurses’ professional quality of life. Future research is needed to further understand the behavioural mechanisms for these relationships, explore other novel mediators and to evaluate the effects of interventions that improve professional quality of life, sleep quality and health among nurses.

## CONFLICT OF INTEREST

The author(s) declare that they have no conflict of interest.

## ETHICAL APPROVAL

The Office of Human Subjects Research Protections approved this study at the National Institutes of Health, Clinical Center (OHSR#13263).

## Supporting information

Fig S1

## Data Availability

The data that support the findings of this study are available from the corresponding author upon reasonable request.
