# Supplementary material for: Professional quality of life, sleep disturbance and health among nurses: A mediation analysis
Source: Nurs Open. 2021 Jul 22;9(6):2771–80. doi: 10.1002/nop2.978 (PMC9584477; doi:10.1002/nop2.978)
Supplement: Supplementary file 1 — Fig S1 [file NOP2-9-2771-s001.pdf]

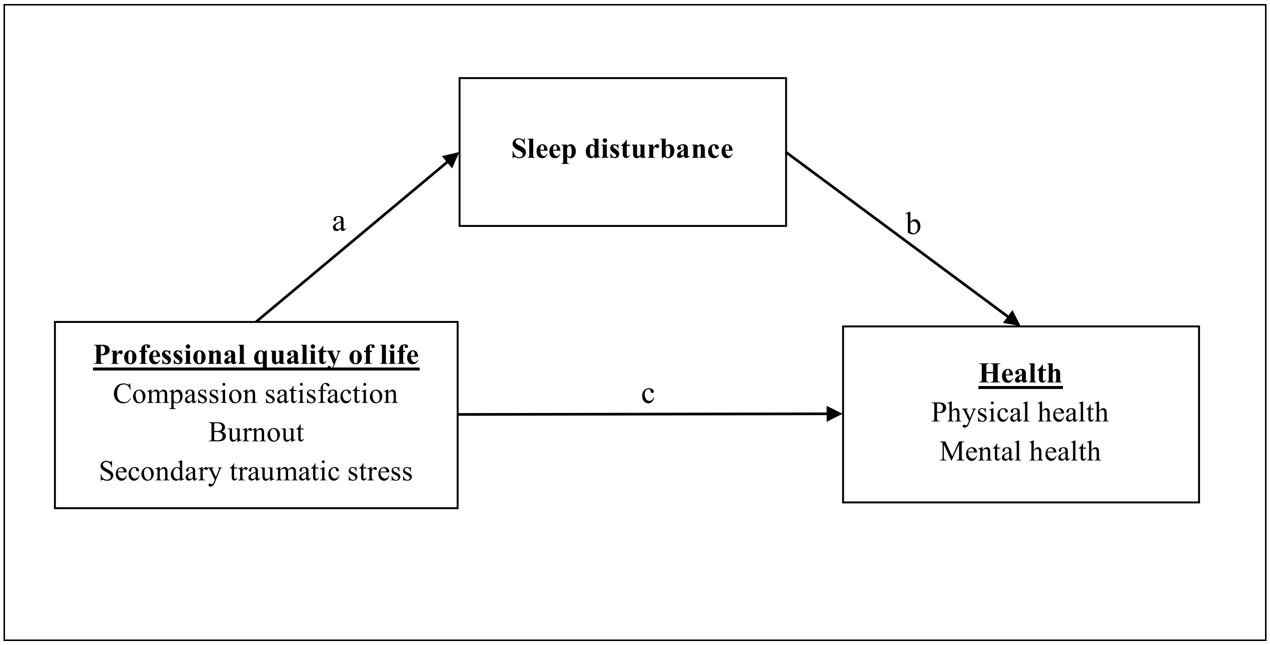

Note. The dependent variable results from the direct effect of the independent variable (path c) as well as the mediating variable (path b). Variable mediating the independent variable can also be involved (path a).
